# Characterization of a caleosin expressed during olive (*Olea europaea *L.) pollen ontogeny

**DOI:** 10.1186/1471-2229-11-122

**Published:** 2011-08-31

**Authors:** Krzysztof Zienkiewicz, Agnieszka Zienkiewicz, María Isabel Rodríguez-García, Antonio J Castro

**Affiliations:** 1 Department of Biochemistry, Cellular and Molecular Biology of Plants, Estación Experimental del Zaidín, Consejo Superior de Investigaciones Científicas (CSIC), Profesor Albareda 1, 18008, Granada, Spain; 2 Department of Cell Biology, Institute of General and Molecular Biology, Nicolaus Copernicus University, Gargarina 9, 87-100, Toruń, Poland; 3 Chair of Plant Physiology and Biochemistry, Institute of General and Molecular Biology, Nicolaus Copernicus University, Gargarina 9, 87-100, Toruń, Poland

## Abstract

**Background:**

The olive tree is an oil-storing species, with pollen being the second most active site in storage lipid biosynthesis. Caleosins are proteins involved in storage lipid mobilization during seed germination. Despite the existence of different lipidic structures in the anther, there are no data regarding the presence of caleosins in this organ to date. The purpose of the present work was to characterize a caleosin expressed in the olive anther over different key stages of pollen ontogeny, as a first approach to unravel its biological function in reproduction.

**Results:**

A 30 kDa caleosin was identified in the anther tissues by Western blot analysis. Using fluorescence and transmission electron microscopic immunolocalization methods, the protein was first localized in the tapetal cells at the free microspore stage. Caleosins were released to the anther locule and further deposited onto the sculptures of the pollen exine. As anthers developed, tapetal cells showed the presence of structures constituted by caleosin-containing lipid droplets closely packed and enclosed by ER-derived cisternae and vesicles. After tapetal cells lost their integrity, the caleosin-containing remnants of the tapetum filled the cavities of the mature pollen exine, forming the pollen coat. In developing microspores, this caleosin was initially detected on the exine sculptures. During pollen maturation, caleosin levels progressively increased in the vegetative cell, concurrently with the number of oil bodies. The olive pollen caleosin was able to bind calcium *in vitro*. Moreover, PEGylation experiments supported the structural conformation model suggested for caleosins from seed oil bodies.

**Conclusions:**

In the olive anther, a caleosin is expressed in both the tapetal and germ line cells, with its synthesis independently regulated. The pollen oil body-associated caleosin is synthesized by the vegetative cell, whereas the protein located on the pollen exine and its coating has a sporophytic origin. The biological significance of the caleosin in the reproductive process in species possessing lipid-storing pollen might depend on its subcellular emplacement. The pollen inner caleosin may be involved in OB biogenesis during pollen maturation. The protein located on the outside might rather play a function in pollen-stigma interaction during pollen hydration and germination.

## Background

In Angiosperms, stamens are the floral organs where pollen development occurs. Each stamen typically consists of a stalk (i.e. the filament) and a bilobed anther with four pollen sacs or microsporangia [1]. In a cross-section of an anther, three distinct compartments are distinguishable: the anther wall (i.e. sporophytic tissues), the locules and the developing pollen grains (i.e. male gametophytes). The anther wall comprises both the connective tissue and four locule-surrounding cell layers namely, from outside to inside, epidermis, endothecium, middle layers and tapetum. The tapetal cells synthesize and secrete several different compounds to the locular space including nutrients, metabolites and wall precursors, in order to promote and regulate pollen development [1]. During anther development, the tapetum undergoes programmed cell death (PCD) and become a lipoidal mass that is deposited coating the pollen wall surface [2]. Therefore, the anther locular fluid represents the chemical link between the anther wall and the symplastically isolated pollen grains [3]. Pollen development begins when pollen mother cells (PMC) divide by meiosis to form tetrads of haploid microspores, which are enclosed by a callose wall. After callose degradation by a tapetal β-1,3-glucanase [4], microspores are released and undergo mitosis to produce bicellular pollen grains. Each pollen grain comprises a large vegetative cell that enclosed a smaller generative cell, which divides to form two sperm cells. During pollen maturation, the vegetative cell accumulates high amounts of storage compounds, which will be further used for pollen germination and pollen tube early growth [5,6].

Neutral lipids constitute the primary energy source in many eukaryotic cells [7]. In plant tissues, they are confined to detached spherical drops called oil bodies (OBs) [8]. Oil bodies have often been regarded as simple storage sites that support periods of active metabolism in the cell [7], however recent data suggest that these organelles are involved in a plethora of dynamic subcellular processes such as lipid trafficking and turnover, and calcium-mediated signalling [9,10]. Structurally, OBs have been proposed to consist of a hydrophobic core containing neutral lipids, such as triacylglycerols (TAGs) and sterol esters, surrounded by a monolayer of amphipatic phospholipids (PLs) embedded with a few unique proteins, namely oleosins, caleosins and steroleosins [11-14].

Caleosins belong to a large gene family found ubiquitously in higher plants and in several lipid-accumulating fungi [7]. Three structural features are common to all caleosins: a well conserved EF-hand, calcium-binding motif [11], a central hydrophobic region with a potential lipid-binding domain, and a C-terminal end with several putative phosphorylation sites. Caleosins are located on the surface of lipid bodies or associated with an ER-subdomain [15]. These proteins are thought to be involved in signal transduction via calcium binding or phosphorylation/dephosphorylation in processes such as membrane expansion, lipid trafficking and OB biogenesis and mobilization [9,10]. Linoleate moieties (18:2) of OB-derived TAGs are oxygenated to (9Z, 11E, 13S)-13-hydroperoxy octadeca-9,11-dienoic acid (13-HPOD) by a specific lipoxygenase [16,17]. It has been hypothesized that 13-HPOD might be reduced to (9Z, 11E, 13S)-13-hydroxy octadeca-9,11-dienoic acid (13-HOD), presumably by the peroxygenase activity of the OB caleosin [18], and released to the cytoplasm [19,20]. Moreover, caleosin expression seems to be up-regulated by both biotic and abiotic stress factors, so these proteins might be involved in oxylipin metabolism [21-23].

Several olive (*Olea europaea *L.) organs and tissues have been reported to contain large amounts of storage lipids [24-28]. The pollen grain is the second most active site, after the seed, in TAG biosynthesis [6,29]. The tapetal cells of the olive anther produce cytosolic lipidic structures, termed pro-orbicules (or pro-Ubisch bodies), as well as a unique type of plastidial lipid bodies, called plastoglobuli [24,30]. The pro-orbicules are secreted by exocytosis to the anther loculus and contain the acyl precursors that are necessary to synthesize the pollen exine. Plastoglobuli are released to the loculus after tapetal cells undergo PCD [2]. These lipidic structures cover the exine to form the outermost layer of pollen grains, the pollen coat, which has important functions in pollination [6]. The mature pollen grain also accumulates a high number of OBs in the cytoplasm of the vegetative cell [24,26,27]. During pollen hydration, these organelles polarize near the aperture through which the pollen tube emerges [27]. Then, OBs are gradually mobilized within the pollen tube during its germination and growth [31]. These data suggest that OBs might provide the pollen grain with energy for a rapid growth of the pollen tube at the early stages of germination in the stigma [32].

In a previous paper, an OB-associated caleosin of about 30 kDa was identified in the mature olive pollen [31]. The apparent synchronicity between the expression pattern of this protein and the dynamics of OBs suggest that it might have a role in the mobilization of storage lipids, as well as in the reorganization of membrane compartments, during pollen germination [31]. Despite the presence of high amounts of lipidic structures in the tissues of the olive anther, little is known about their biogenesis, and no data regarding the presence and function of caleosins during anther development have been published to date. The present paper is the first report about the cellular localization and expression pattern of a caleosin in the anther of an oil storing species like olive (*Olea europaea *L.). The putative function of this caleosin in the context of olive sexual reproduction is discussed.

## Results

### OBs behaviour during olive anther development

**Figure 1:**
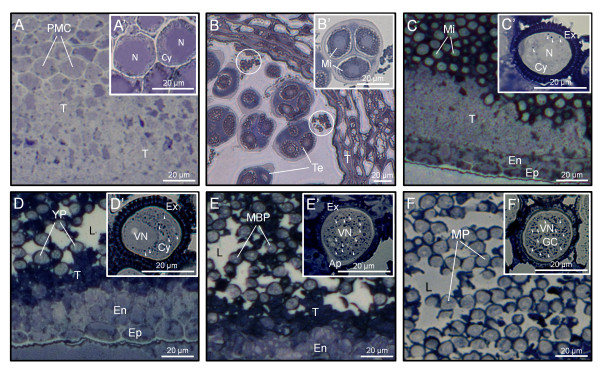
**Sudan black B staining of neutral lipids in sections from olive anthers**. Light microscopy sections (A-F) -and enlarged views (A'-F')- of olive anthers at the PMC (A and A'), Te (B and B'), Mi (C and C'), YP (D and D'), MBP (E and E') and MP (F and F') stages. Oil bodies are indicated by arrowheads, while circles denote lipidic masses. Ap: pollen aperture; Cy: cytoplasm; En: endothecium; Ep: epidermis; GC: generative cell; L: anther locule; MBP: mid bicellular pollen grain; Mi: microspore; MP: mature pollen grain; N: microspore nucleus; PMC: pollen mother cell; T: tapetum; Te: tetrad; VN: vegetative nucleus; YP: young pollen grain.

The Sudan black B technique was used to study OB distribution and behaviour during anther ontogeny. At early stages of development, when anthers contain PMCs, the content of neutral lipids was very low and no OBs were observed in either PMCs or any other tissue of the anther (Figure 1A, A'). At tetrad stage, the anther tissues appeared faintly stained, while the anther locule contained lipidic masses of granular appearance (Figure 1B). The tapetum edge facing the loculus also appeared densely stained. After microspores were released from tetrads, we found a significant increase in the lipoid material present in the tapetal cells, as well as in the anther locule, which was densely stained (Figure 1C). At this stage, very few OBs were distinguished scattered in the cytoplasm of developing microspores, whereas the exine appeared heavily stained (Figure 1C'). Upon microspore first mitosis, the tapetal tissue, with apparent symptoms of degeneration, contained high amounts of neutral lipids as demonstrated by the intense staining (Figure 1D). At this stage, the locular fluid was not uniformly stained and lipids formed patches mainly distributed in the vicinity of developing pollen grains. A significant increase in the number of OBs was observed in the cytoplasm of young pollen grains, while the pollen wall was also greatly stained (Figure 1D'). During pollen maturation steps, the number of OBs present in the cytoplasm of the vegetative cell progressively increased (Figure 1E'), while the amount of neutral lipids in the anther locule decreased and formed densely stained patches (Figure 1E). Just before anthesis, the remnants of the tapetum were still densely stained. Lipids in the anther locule decreased significantly and were mostly coating the pollen wall (Figure 1F). At this stage, the cytoplasm of mature pollen grains was filled up with numerous OBs (Figure 1F').

### Expression of caleosin during the olive anther development

**Figure 2:**
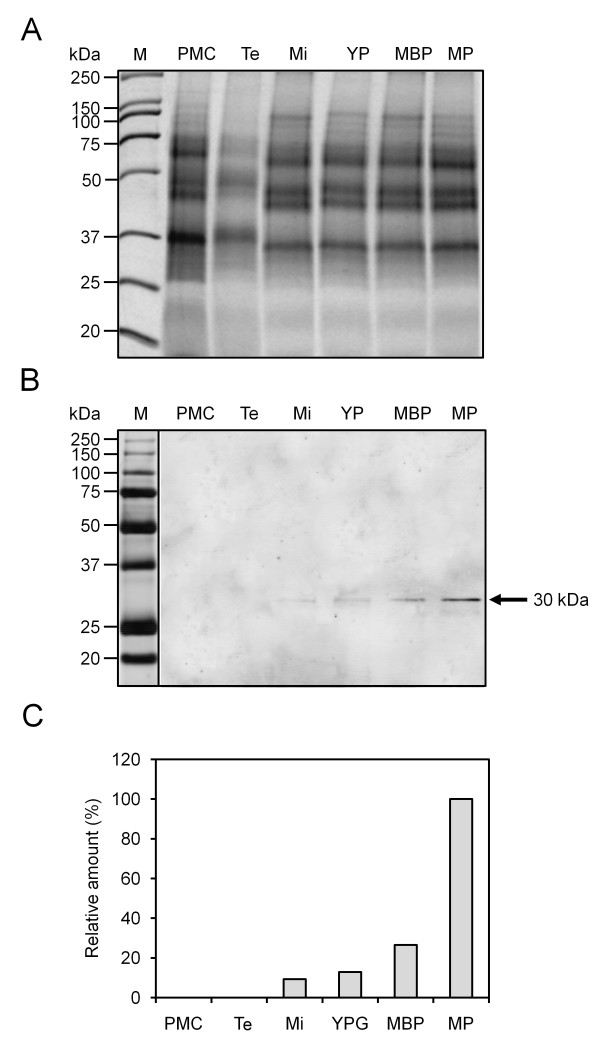
**Caleosin expression pattern during olive anther development**. (A) Coomassie-stained gel of total proteins from olive anthers at the pollen mother cell (PMC), tetrad (Te), microspore (Mi), young pollen grain (YP), mid bicellular pollen grain (MBP) and mature pollen grain (MP) stages. (B) Western blot as in figure 2A probed with a FL anti-Clo3 Ab, followed by an anti-rabbit IgG Alexa 633-conjugated secondary Ab. A band of about 30 kDa (arrow) was recognized by the FL Ab. (C) Densitometric data corresponding to the 30 kDa band from figure 2B.

Western blot experiments showed the presence of a band of about 30 kDa in the developing anther of olive, which was cross-recognized by the FL Ab (Figure 2). The specificity of this Ab was demonstrated in a previous work [31]. Thus, a faint protein band was hardly detected after meiosis. Caleosin levels significantly increased after the asymmetric mitosis of microspore and during the subsequent steps of pollen maturation (Figure 2B). No protein could be detected in early stages of anther development. Densitometric data showed that the highest expression of caleosin occurred at pollen maturity, just before flower anthesis and anther dehiscence (Figure 2C).

### Immunolocalization of caleosin in the anther tissues

**Figure 3:**
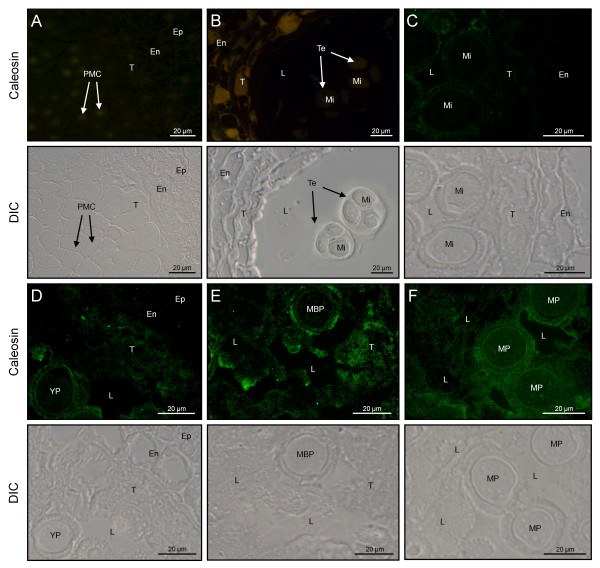
**Fluorescence microscopy localization of caleosin in the olive anther**. Sections from olive anthers at the PMC (A), Te (B), Mi (C), YP (D), MBP (E) and MP (F) stages were incubated with a FL anti-clo3 Ab, followed by an anti-rabbit IgG-Alexa Fluor 488-conjugated secondary Ab. Differential interference contrast (DIC) images of serial sections were also obtained to better visualize the different tissues of the anther. En: endothecium; Ep: epidermis; L: anther locule; MBP: mid bicellular pollen grain; Mi: microspore; MP: mature pollen grain; PMC: pollen mother cell; T: tapetum; Te: tetrad; YP: young pollen grain.

The cellular localization of the 30 kDa-caleosin in the anther tissues was examined during its development by both fluorescence and transmission electron microscopy. In young anthers containing microsporocytes, the lack of fluorescent signal in the anther indicated that caleosin was absent at that point of development (Figure 3A). Caleosin was first detected at the free microspore stage. The protein was located in both the tapetum and the locular fluid, as well as in the exine layer, whereas the cytoplasm of the developing microspore was devoid of significant fluorescent labelling (Figure 3B). After the mitotic division of microspores, caleosin levels increased in the tapetal tissue (Figure 3C). At this stage, a spotted fluorescent pattern started to be visible in the cytoplasm of the pollen grain (Figure 3C). During pollen maturation, patches of fluorescent material were inconsistently distributed in the anther locule, being mainly located in the vicinity of developing pollen grains (Figure 3D) Moreover, the intensity of fluorescence increased in both the cell wall and the cytoplasm of the pollen grain. At the end of pollen maturation, the fluorescent labelling was highest in the material adhered to the pollen wall and inside the cytoplasm of the vegetative cell (Figure 3E). In the degenerated tapetum, a significant reduction of the fluorescent signal was observed.

**Figure 4:**
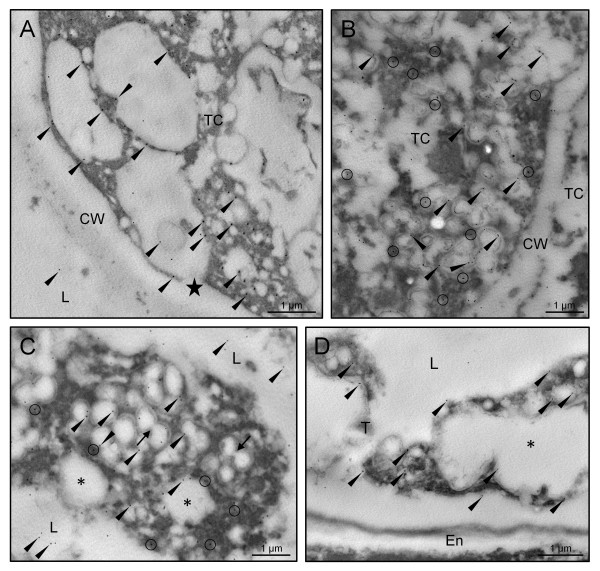
**Transmission electron microscopy localization of caleosin in the olive tapetum**. Sections from olive anthers at the young microspore (A), vacuolated microspore (B), young pollen grain (C) and mature pollen grain (D) stages were incubated with a FL anti-Clo3 Ab, followed by an anti-rabbit IgG-15 nm gold conjugated secondary Ab. Oil body-associated caleosin is indicated by arrowheads, while circles denote ER-associated caleosin. Note that: i) the tapetal cell vesicles fused with the plasma membrane (star), releasing their content to the loculus, and ii) oil bodies merged (arrows) to produce larger OBs and lipoid masses (asterisks). CW: cell wall; En: endothecium; L: anther locule; TC: tapetal cell; T: tapetum.

Gold immunolabelling of the 30 kDa caleosin in sections of olive developing anthers provided additional details about its subcellular localization, and confirmed the tissue distribution pattern observed by fluorescence microscopy. At the young microspore stage, gold particles were located on the boundaries of the numerous vesicles that filled the cytoplasm of tapetal cells. These vesicles fused with the cell membrane and released their content to the anther locule and the tapetal intercellular space (Figure 4A). After microspore vacuolization and just prior to mitosis, caleosins were found on the surface of lipid droplets that filled up the cytoplasm of tapetal cells, as well as on the closely associated ER cisternae (Figure 4B). Upon microspore mitosis, the tapetal cells underwent PCD and lost their integrity. Caleosins were detected on the boundaries of lipid droplets embedded in electron-dense material and surrounded by ER cisternae, which contained gold particles attached to their surface (Figure 4C). Lipid droplets merged (Figure 4C, arrows) and also showed gold labelling on their shells. At the end of pollen maturation, the tapetum was reduced to large oil drops with patches of an electron-dense material, which still showed some gold particles attached (Figure 4D).

**Figure 5:**
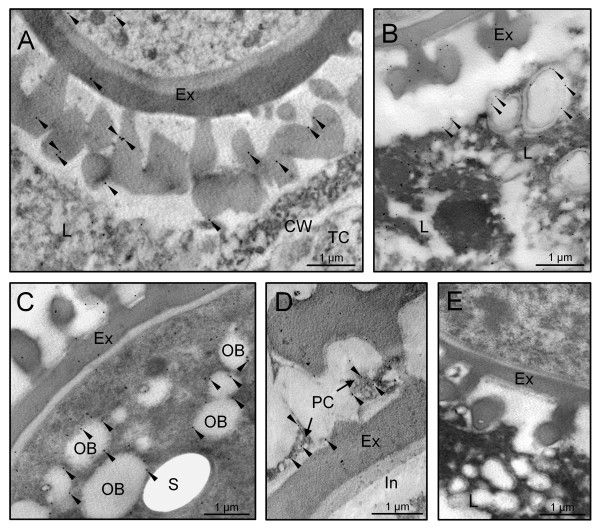
**Transmission electron microscopy localization of caleosin in the olive pollen and locular fluid**. Sections from olive anthers at the mid microspore (A), young pollen grain (B), mid bicellular pollen grain (C) and mature pollen grain (D) stages were treated as in figure 4. Gold labelling is indicated by arrowheads. (E) Control reaction by omitting the FL anti-Clo3 Ab showing the absence of gold labelling. CW: cell wall; Ex: exine; In: intine; L: anther locule; OB: oil body; PC: pollen coat; S: starch; TC: tapetal cell.

**Figure 6:**
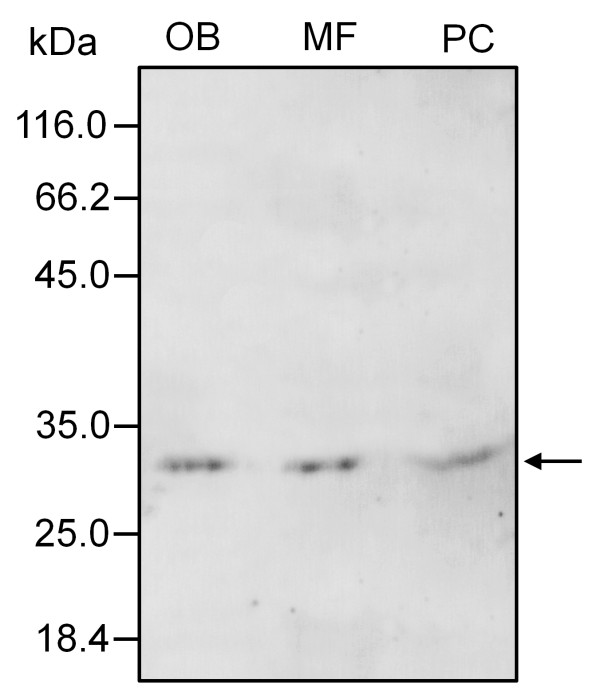
**Subcellular distribution of caleosin in the olive pollen grain**. Immunoblots were probed with a FL anti-Clo3 Ab, followed by an anti-rabbit IgG Alexa 633-conjugated secondary Ab. A single band of about 30 kDa was detected in all pollen fractions (arrow). MF: microsomal fraction; OB: oil body fraction; PC: pollen coat proteins.

At the early steps of microspore development, a few gold particles were also located in the exine sculptures, as well as associated with short ER cisternae scattered in the cytoplasm of developing microspores (Figure 5A). Caleosins were deposited on the forming exine together with an electron-dense material (Figure 5A). As microspore vacuolization progressed, the number of gold particles was significantly higher in both the exine layer and the microspore cytoplasm (data not shown). Upon bicellular pollen formation, the presence of caleosins in the exine layer increased (Figure 5B). In the anther locule, gold particles appeared attached to the boundaries of both oil bodies and ER cisternae. As pollen maturation progressed, the number of gold particles increased (Figure 5C and 5D). The signal was localized on the boundaries of the numerous OBs scattered in the cytoplasm of the vegetative cell and associated with ER cisternae (Figure 5C). In addition, caleosins were found in the electron-dense debris derived from the tapetum that filled the cavities of the exine (Figure 5D). The intine, the vegetative nucleus and the generative cell were devoid of gold particles. Control reactions were performed by omitting the primary Ab and did not show any significant labelling (Figure 5E). Upon subcellular fractionation, Western blot analyses confirmed the presence of a unique caleosin in pollen OBs and the microsomal fraction, as well as in the pollen coat (Figure 6).

### Calcium binding ability of olive pollen caleosin

**Figure 7:**
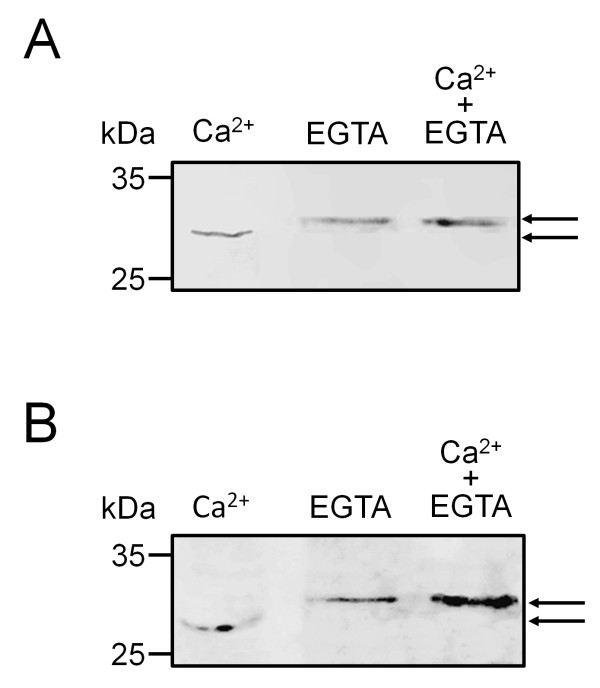
**Effects of calcium ions on electrophoretic mobility of olive pollen caleosin**. Effects of calcium ions on electrophoretic mobility of caleosin isolated from pollen OBs (A) and pollen coat (B). Proteins were extracted from pollen OBs and pollen coat and pre-treated with EGTA. Ten μg of proteins were incubated with CaCl_**2 **_(left lane), CaCl_**2 **_followed by EGTA (middle lane), or both CaCl_**2 **_and EGTA simultaneously (right lane). Immunoblots were probed with a FL anti-Clo3 Ab, followed by an anti-rabbit IgG Alexa 633-conjugated secondary Ab. Arrows show caleosin bands. Protein markers (kDa) are indicated on the left.

The capacity of pollen caleosin to bind calcium *in vitro *was tested. Caleosins were isolated from both OBs and the pollen coat and treated with EGTA to remove endogenous Ca^2+^. Then, caleosins were electrophoresed and immunodetected by Western blot. In both pollen-purified OBs (Figure 7A) and pollen coat extracts (Figure 7B), caleosin migrated faster after Ca^2+ ^treatment. Accordingly, the migration of the Ca^2+^-linked caleosin was retarded by EGTA treatment. No mobility shift was found after incubation with other cations like Mg^2+ ^or K^+ ^(data not shown).

### Structural conformation of olive pollen caleosin

**Figure 8:**
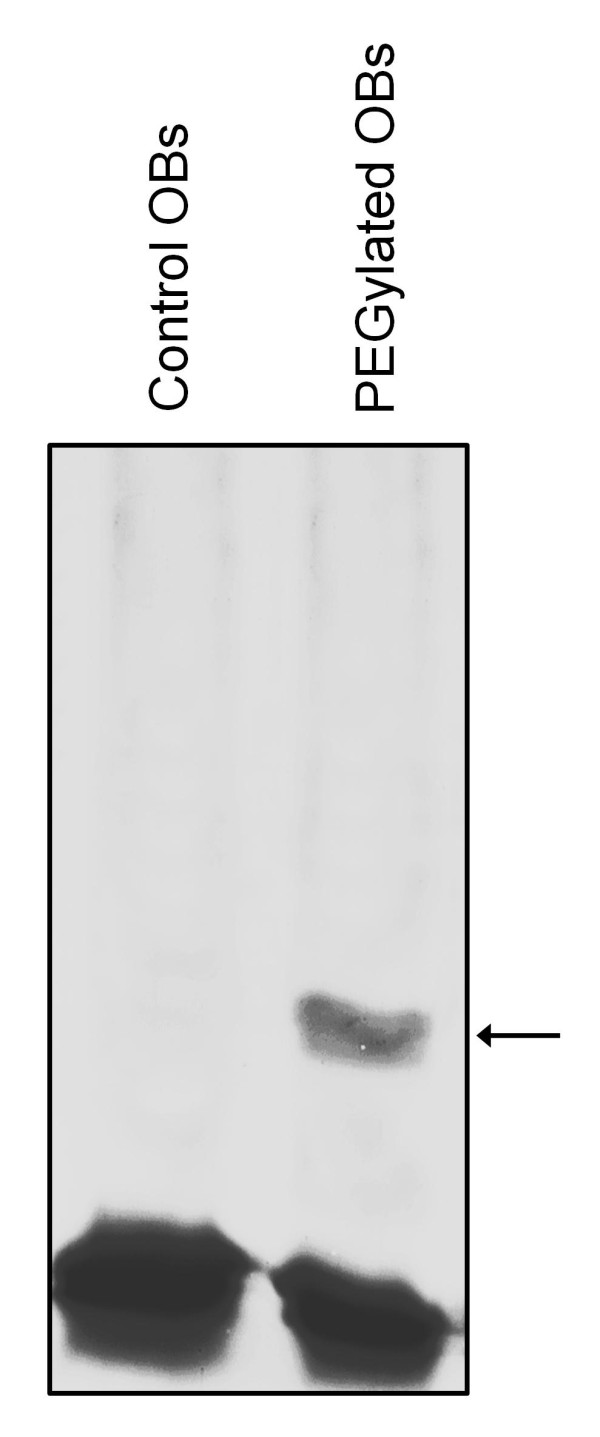
**PEGylation of olive pollen OBs and immunodetection of caleosin by Western blotting**. Oil bodies were isolated from olive pollen, incubated with PEG-MAL (5,000 Da), run on a 7.5% polyacrylamide Bis-Tris gel and transferred onto a PVDF membrane. Oil body-associated caleosin was immunodetected using an αN anti-Clo3 Ab, followed by an anti-rabbit IgG-DyLight 549 conjugated secondary Ab. One prominent higher molecular weight band (arrow), which corresponds to one modified Cys residue, was visible in PEGylated OBs but not in the control (i.e. non-PEGylated OBs).

To examine whether the N- and C-terminal domains of the olive pollen OB-associated caleosin are exposed to the cytosol, we performed PEGylation assays with isolated OBs using the 30 kDa caleosin as OB marker. For this purpose, a membrane impermeable probe (PEG-MAL, 5,000 Da) that reacts with sulfhydryl groups of Cys residues was used. The PEG-MAL adds 5 kDa for each SH blocked, so that PEGylated OBs migrate slower than non-PEGylated OBs in SDS-PAGE. Thus, the number of higher mass molecular bands in SDS-PAGE should correspond to the number of Cys present in the protein. In our experiments, a single prominent band of higher molecular mass was observed, indicating that only one Cys residue reacted with PEG-MAL (Figure 8).

**Figure 9:**
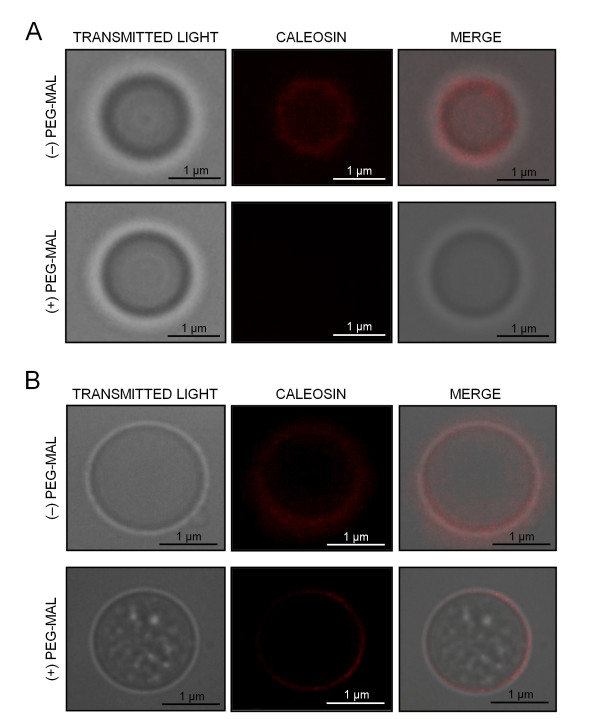
**PEGylation of olive pollen OBs and localization of caleosin by fluorescence microscopy**. (A) Localization of caleosin on PEGylated (+) and non-PEGylated (-) OBs from pollen using a FL anti-Clo3 caleosin Ab, followed by a secondary Ab conjugated with DyLight 549. After PEGylation, the accessibility of the FL anti-Clo3 Ab was hampered and no fluorescence was observed. (B) Localization of caleosin as above but using an N-terminal (αN) anti-Clo3 caleosin Ab. After PEGylation, the primary Ab was able to bind to caleosin.

In parallel, we carried out immunolocalization experiments in both PEGylated and non-PEGylated OBs, in order to check the accessibility of two anti-Clo3 antibodies (Figure 9). The FL Ab was able to recognize a caleosin on the surface of non-PEGylated OBs, being visualized as a red fluorescent labelling (Figure 9A, upper panel). PEGylation impeded the binding of the FL Ab to the protein, leading to the loss of fluorescence (Figure 9A, lower panel). Similarly, the αN Ab bound to the caleosin and produced a red fluorescent signal (Figure 9B, upper panel). However, PEGylation did not hamper the binding of the αN Ab to the caleosin (Figure 9B, lower panel). These results suggest that: i) the FL Ab specifically recognizes the C-terminal domain of the protein, and ii) both the C- and the N-terminal domains of the protein are exposed to the cytosol.

## Discussion

The anther tapetum is the main supplier of nutrients and cell wall precursors for developing pollen grains [6]. This tissue accumulates high amounts of lipidic material during its development, and any anomaly in this process leads to defects in both tapetum morphology and pollen exine ontogenesis [33]. The tapetal cells of the olive anther lacked typical seed OBs [8]. However, at the mid bicellular pollen stage, tapetal cells showed numerous lipid-containing droplets, closely packed and enclosed by ER-derived tubules and vesicles. These structures resembled very much those termed tapetosomes [34], which were described in the tapetum of species of the *Brassicaceae *family [34-37]. Yet, these structures in olive showed some morphological differences when compared with the *Brassicaceae *tapetosomes. For instance, lipid droplets in olive were not visualized as electron-dense structures, not even after osmium fixation [27,38], likely due to differences in their lipidic composition. This fact allowed us a better visualization of the associated ER membranes. Moreover, tapetosomes from *Brassicaceae *were described as discrete entities [34,39], while the structures reported in the olive showed unclear outlines. Such structural differences could be explained by methodological (i.e. sample processing) discrepancies, since tapetosomes are osmotically active organelles [34].

The olive tapetum is of the parietal type, and the tapetal cell walls begin to disintegrate when microspores are released from the tetrad [24]. After completion of olive tapetum degradation, the structures containing both lipid droplets and ER-vesicles were released to the locule and deposited onto the surface of the developing pollen. At the final stage of maturation, electron-dense remnants of tapetum filled the exine cavities. These results are consistent with previous observations in *Brassica *[40], and suggest that these masses of lipid droplets and ER-vesicles also contribute to pollen coat formation in the olive. In *Brassica *tapetosomes, the composition of lipid droplets is similar to seed oil bodies and contain mainly TAGs and PLs [34,37,41]. These TAGs disappeared in the course of anther maturation [42], at the time that lipid droplets and ER-derived vesicles stored alkanes and flavonoids, respectively [37,40]. We can speculate that something similar might occur in olive, though additional experiments will be necessary to confirm this hypothesis. The earliest evidence concerning tapetosome function showed that both alkanes and flavonoids were discharged to the pollen surface and protected the pollen grain from UV damage and desiccation [40]. Moreover, mutations affecting genes involved in tapetosome biogenesis lead to deficient pollen coat formation and reduced pollen fertility [43].

Current data about the presence and function of caleosins in the reproductive tissues of higher plants are very scarce. In a previous paper, we described a novel caleosin from olive pollen, which might be involved in OB mobilization and membrane compartment rearrangements during pollen germination [31]. To our knowledge, the data presented here constitute the first report regarding the expression pattern and cellular localization of a caleosin during anther development in an oil-storing plant species. Using an Ab raised against a heterologous caleosin (Clo3) expressed in *Arabidopsis *[22], a caleosin isoform with a molecular mass of about 30 kDa was detected in the whole anther of olive from the early microspore stage onwards. The specificity of this Ab was demonstrated by immunoprecipitation and sequencing experiments in a previous paper [31]. *In situ *expression and localization studies provided us valuable information about the spatial and temporal distribution of this caleosin in the different compartments of the anther. According to our data, it seems that two different expression programmes affecting a 30 kDa caleosin protein coexist in the olive anther during its development.

The sporophytic programme began soon after microspore release from tetrads. At this stage, the 30 kDa caleosin was mostly expressed in the tapetal cells, so it can be assumed that the protein is first synthesized in the tapetum. These data support the idea that caleosins from the exine layer and the pollen coat have a sporophytic origin, as previously suggested [31]. At this early stage, caleosins were specifically located at the boundaries of numerous vesicles of different sizes spread throughout the cytoplasm of tapetal cells, which subsequently discharge their content to the anther locule. Caleosins also appeared in the tectum and bacula, which constitute the sculpted layer of microspore exine. Interestingly, the presence of caleosins in the exine innermost layer (i.e. nexine) at the stage of bicellular pollen was quite remarkable. This finding suggests that caleosins might move from the outermost edge to deeper layers of the pollen exine. A few proteins have been described in *Brassicaceae *tapetosomes, including a 45 kDa oleosin that coats lipid droplets [34], as well as two proteins, named calreticulin and luminal binding protein (BiP), which are located in the luminal space of ER-derived vesicles [44]. Here, we demonstrated that a caleosin was also located on the boundaries of both lipid droplets and ER cisternae in olive tapetal cells. In *Brassica*, the 45 kDa oleosin underwent selective proteolysis and a 37 kDa fragment was further released to the anther loculus and retained in the pollen coat upon tapetum degradation [37]. In contrast, the olive tapetum caleosin did not undergo proteolysis and was retained fully intact in the pollen coat. Similarly, caleosins were also found in the pollen coat of *Brassica napus *[45] and *Arabidopsis thaliana *[46]. Our data were also consistent with the idea that tapetum lipid droplets are assembled in, and then detached from the ER [44], and suggest that caleosins might participate in their biogenesis. The pollen coat is a site for functional proteins involved in cell wall loosening, pollen hydration and pollen-stigma communication [45-47]. The function of pollen coat-associated caleosins is currently unknown, but the presence of several phosphorylation sites, as well as a lipid-binding domain and a single EF-hand Ca^2+^-binding domain, suggests that these proteins might have a function in pollen-stigma signalling [45].

The gametophytic programme started during microspore maturation. The absence or reduction in the machinery for translation at the tetrad and early microspore stages might explain the lack of significant levels of caleosin at these developmental stages. Thus, the ribosomal population significantly decreased after meiosis [48,49]. Moreover, the low levels of caleosins observed in the young microspore correlated well with the ER shortage at this stage [50]. Mature olive pollen contains numerous OBs, whose number increased from the late microspore stage onwards [27, the present work]. Interestingly, the expression levels of the 30 kDa caleosin in the developing pollen increased in parallel with the OB number. These results suggest that the synthesis and targeting of this caleosin is tightly connected with OB biogenesis in the pollen grain. Purified 30 kDa caleosin from olive pollen was shown to bind Ca^2+ ^*in vitro*, as was demonstrated for its counterpart in seeds [9,12,18,21]. Calcium binding regulates peroxygenase activity of caleosins [18] and it might mediate OB-vacuole interactions that affect mobilization of OBs during seed germination [10]. Similarly, it has been suggested that Ca^2+ ^might also regulate caleosin-mediated OB mobilization and membrane compartment reorganization in the growing pollen tubes [31]. Therefore, caleosins might also be involved in Ca^2+^-mediated fusion of nascent lipid microbodies to produce mature larger oil bodies in the pollen grain.

Experimental data about how caleosin expression is regulated in plant tissues are scarce to date. A few studies in *Arabidopsis *showed that AtCLO3 caleosin expression is up-regulated by abscisic acid (ABA) in biotic and abiotic stress responses [21-23], whereas AtCLO4 might be a negative regulator of ABA responses [51]. In the anther, ABA accumulates in the sporogenous and tapetal cells, but is undetectable in the microspores/pollen grains [52]. This is in good agreement with recent studies indicating that ABA might regulate cell separation during early anther development [53], as well as apoplastic sugar transport in the tapetum [54]. The possibility that ABA regulates caleosin expression in the olive anther is currently under investigation in our laboratory.

Caleosins are associated with different membranes in plant cells. In *Arabidopsis*, AtCLO3 was localized on microsomal membranes, the chloroplast envelop, and the vacuole tonoplast of leaves [22,55]. Protease protection assays suggest that AtCLO3 caleosin is an integral membrane protein arranged in a type-I orientation [56], with the N-terminal domain facing the lumen and the C-terminal domain facing the cytosolic side [22]. This orientation is typical of a protein involved in signal transduction or regulation within cells, and agrees well with its putative role in oxylipin signalling during biotic and abiotic stress responses. The olive pollen caleosin is also firmly bound to the ER cisternae, the plasmalemma and the vacuole membrane [31, the present work]. Similarly, we can speculate that this caleosin has a type I membrane orientation, although further experimental work will be necessary to confirm this hypothesis. Contrary to AtCLO3, the olive pollen 30 kDa caleosin was also found tightly associated with purified OBs even after vigorous and repeated washes. It was suggested because of thermodynamic reasons that OB-bound caleosins expose both their N- and C-terminal domains to the cytosol, while the central hydrophobic domain remains buried within the PL monolayer [12]. Protease protection assays showed that about 1 and 2 kDa of the N- and C-terminal domains, respectively, are accessible to proteolytic attack [18,22,57]. The alignment of caleosin amino acid sequences from several plant species showed the presence of a single conserved Cys in the C-terminal domain of the protein [12,15]. On this assumption, we carried out PEGylation experiments in order to validate this model in the olive pollen OB-associated caleosin. PEG-MAL is a membrane-impermeable probe that reacts irreversibly with sulfhydryl groups of Cys, adding 5 kDa for each SH group. This is a simple and helpful method to localize Cys residues in regions of membrane proteins oriented toward the cytosolic side [58]. Accordingly, after PEG-MAL treatment of intact isolated OBs, we found an additional higher mass band in SDS-PAGE gels, as expected if a single Cys residue is exposed to the cytosol. These results were confirmed at the microscopic level by testing the accessibility of different anti-caleosins Abs. Our data strongly suggest that: i) Cys PEGylation does not hamper the binding of the αN Ab to the caleosin but impedes the binding of the FL Ab, and ii) the FL Ab only recognizes the C-terminal part of the protein. Therefore, our findings here showed that the olive pollen OB-bound caleosin expose both their C- and N-terminal domains to the cytosol, so its structural conformation could be similar to that of caleosins from seed OBs.

## Conclusions

To date, very little is known about caleosin with regard to its biological function during sexual reproduction in plants. In a pioneer work, we identified and characterized a new caleosin from olive pollen, which may be involved in OB mobilization and membrane compartment rearrangement during pollen germination. In this paper, we reported for the first time a caleosin expressed in the tapetum and developing pollen of an oil-storing plant species like olive. We found that a caleosin of about 30 kDa is synthesized in both the tapetum and developing pollen, but its synthesis is independently regulated. Moreover, the olive pollen caleosin has a dual sporophytic and gametophytic origin. The tapetum-derived caleosin is deposited intact onto the pollen exine and as part of the pollen coat. The intrinsic characteristics of this protein suggest that its function in this place might be associated with pollen-stigma interactions during pollen hydration and germination. The pollen inner caleosin is synthesized by the vegetative cell. Our data connect the synthesis and targeting of this caleosin with OB biogenesis in both the tapetum and the pollen grain. Thus, caleosins may be involved in Ca^2+^-mediated fusion of nascent lipid microbodies to produce mature larger OBs. In brief, the results indicate that caleosins may have a relevant function in the early steps of the sexual reproductive process in those species possessing lipid-storing pollen.

## Methods

### Plant material

Developing anthers and mature pollen grains were collected from *Olea europaea *(L.) trees (cv. Picual) grown in the province of Granada (Spain). Mature pollen grains were harvested from dehiscent anthers by vigorous shaking of flowering shoots inside large paper bags. Then, pollen samples were sieved through an appropriate set of meshes to remove floral debris. Anthers at the stages of PMC, tetrad, free microspore, young bicellular pollen, mid bicellular pollen and mature pollen were dissected from floral buds under a stereomicroscope (Zeiss, Germany), and staged by squashing and DAPI (4',6-diamidino-2-phenylindole) staining. The material was processed freshly or frozen in liquid N_2 _and stored at -80°C until use.

### Sample preparation for microscopy analysis

Anthers at different stages of development and mature pollen grains were fixed with 4% (w/v) paraformaldehyde and 0.2% (v/v) glutaraldehyde in 0.1 M cacodylate buffer (pH 7.2) for 24 h at 4°C. Then, samples were dehydrated in ethanol series and embedded in Unicryl resin (BBInternational, UK) according to [31]. Semi-thin (1 μm) sections were obtained with a Reichert-Jung Ultracut E microtome (Leica Microsystems, Germany) using a glass knife, and placed on BioBond™ (BBInternational) coated slides. Ultra-thin (80 nm) sections were also cut using a diamond knife (Diatome, USA) and mounted on formvar coated nickel grids.

### Neutral lipids staining

The technique of Sudan Black B was used for staining neutral lipids in developing olive anthers, following the method described by [59]. Semi-thin sections were stained at 60°C for 30 min using a fresh saturated solution of Sudan Black B, prepared in 70% (v/v) ethanol. Observations were carried out with an Axioplan microscope (Carl Zeiss, Germany) and images were recorded with a ProGres C3 digital camera using the ProGres CapturePro v2.6 software (Jenoptik AG, Germany).

### Immunolocalization of caleosins in olive anthers

Slides containing semi-thin sections of olive anthers at different developmental stages were incubated with a solution containing 1% (w/v) BSA in phosphate buffered saline (PBS) solution (pH 7.2) for 1 h at room temperature, in order to block non-specific binding sites. Caleosin was immunodetected by incubation overnight at 4°C with a polyclonal serum (diluted 1:50 in blocking solution) raised against the full length (FL) sequence of Clo3 caleosin from *Arabidopsis thaliana*, following by an anti-rabbit IgG-Alexa Fluor 488-conjugated secondary antibody (Ab) (Molecular Probes, USA) (diluted 1:100 in blocking solution) for 1 h at 37°C. Sections were examined in an Axioplan microscope (Carl Zeiss) equipped with both an epifluorescence system, using the following filter combination: BP450-490, FT510 and LP520. Transmitted light images were captured from serial sections of the resin blocks using a Nomarski optic system. For immunogold experiments, ultra-thin sections were processed as described previously [31]. In control sections, the primary Ab was omitted.

### Purification of OBs from mature olive pollen

Oil body isolation was carried out at 4°C as described by [60], with some modifications. Pollen samples were ground in a homogenization buffer consisting of 100 mM 4-(2-hydroxyethyl)-1-piperazineethanesulfonic acid (HEPES) buffer (pH 7.5) containing 0.4 M sucrose, 10 mM KCl, 1 mM EDTA and 2 mM dithiothreitol (DTT). Homogenates were centrifuged at 6,000*g *for 2 min to remove debris. Then, they were fractionated by centrifugation at 20,000*g *for 20 min. The lower fraction was centrifuged at 100,000*g *for 1 h. The pellet, corresponding to the microsomal fraction, was recovered and stored at -20°C until use. The upper lipid pad was resuspended in 50 mM Tris-HCl buffer (pH 7.2) containing 1 M NaCl, 9 M urea and 0.2% (w/v) Tween 20. The suspension was diluted with 0.5 ml of homogenization buffer and layered with four volumes of 100 mM HEPES buffer (pH 7.5) containing 0.1 M sucrose. Oil bodies were recovered after centrifugation as above and washing steps were performed according to [60]. Finally, the OB fraction was mixed with 0.6 M sucrose in 10 mM PBS buffer (pH 7.5) and stored at -20°C until use.

### Protein extraction

Olive anther samples (approximately 0.1 g each sample) were ground in liquid N_2 _to a very fine powder, which was resuspended in 1.5 ml of extraction buffer [40 mM Tris-HCl (pH 7.4), 2% (v/v) Triton X-100, 1 mg/ml ascorbic acid, 60 mM DTT, 5% (w/v) polyvinylpyrrolidone (PVPP) and 15 μl of a protease inhibitor cocktail (catalogue no. P9599, Sigma-Aldrich, USA)]. After centrifugation at 10,000*g *for 30 min at 4°C, the resulting supernatants were transferred to 15 ml tubes and mixed with 10 volumes of 20% (w/v) trichloroacetic acid (TCA) and 0.2% (w/v) DTT in acetone. Proteins were precipitated at -20°C overnight, and collected by centrifugation at 10,000*g *for 30 min at 4°C. The resulting pellets were rinsed with cold acetone three times for 15 min each, resuspended in sample buffer [61], dispensed into aliquots and stored at -20°C until use.

Pollen samples (0.1 g) were mixed with 1.5 ml of extraction buffer and proteins were eluted by stirring for 6 h at 4°C. The resulting homogenates were processed as above. Pollen coat (PC) protein extracts were prepared as described previously by [62]. Briefly, coating was removed by resuspending 1 g of pollen in 1 ml of cyclohexane. After centrifugation at 14,000*g *for 30 sec, the cyclohexane fraction was transferred to a clean 1.5 ml tube. Lipids were removed by repeated centrifugations at 21,000*g *for 20 min. Then, samples were spun in a Speed-Vac concentrator (Braun Biotech International GmbH, Germany) at 4°C until the solvent was completely evaporated. Pollen coat proteins were resuspended in 1 ml of sample buffer [60] by vigorous vortexing for 20 min.

Purified OBs and microsomal fractions were mixed 1:2 (v/v) with a solution containing 7 M urea, 2 M thiourea, 4% (w/v) 3-[(3-cholamidopropyl) dimethylammonio]-1-propanesulfonate (CHAPS), 3% (w/v) SDS, 60 mM DTT and 0.5% (v/v) Bio-Lyte 3-10 buffer (Bio-Rad, USA), incubated for 1 h at 4°C with occasional vortexing, and processed as above.

The protein concentration in each sample was estimated by using the 2D Quant Kit (Amersham Biosciences, USA) according to the manufacturer's instructions.

### SDS-PAGE and Western blotting

SDS-PAGE was performed according to [60]. Twenty five μg of total protein per sample were loaded on 12% (w/v) polyacrylamide gels and electrophoresed using a Mini-Protean 3 apparatus (Bio-Rad, USA). After electrophoresis, gels were stained with Coomassie Brilliant Blue R250 according to standard procedures. Proteins were electroblotted onto a polyvinylidene fluoride (PVDF) membrane in a Semi-Dry Transfer Cell (Bio-Rad). Immunodetection of caleosin was carried out as previously described [31]. The signal was detected in a Pharos FX molecular imager (Bio-Rad).

### Calcium binding *in vitro *assays

Calcium binding *in vitro *assays were carried out as previously described by [12]. Twenty five μg of total proteins from either pollen OBs or pollen coat were resuspended in 250 μl of 10 mM Tris-HCl buffer (pH 7.5) and mixed with an equal volume of 200 mM ethylene-glycol-bis(β-aminoethylether)-*N, N, N', N'*-tetraacetic acid (EGTA) for 5 min at room temperature. Samples were then incubated with 100 mM CaCl_2 _for 5 min at room temperature, followed by incubation with 100 mM EGTA. Effects of Ca^2+ ^on the caleosin mobility were analyzed by SDS-PAGE and Western blot as described above.

### PEGylation assays

PEGylation assays were performed according to [58] with some modifications. Pollen OBs were purified as described above and treated (250 μl) with a solution containing 0.1 M Tris-HCl (pH 7.0), 10 mM metoxypolyethylenglycol-maleimide (PEG-MAL) 5,000 Da (Sigma-Aldrich) and 1 mM EDTA, for 0, 5 and 20 min, at 4°C in the dark. The PEGylation reaction was stopped by adding an equal volume of Laemmli sample buffer containing 100 mM DTT. OB samples (40 μl each) were loaded on SDS-PAGE Bis-Tris gels (7.5%) and electrophoresis was conducted in a Mini-Protean 3 apparatus (Bio-Rad) using a 3-(N-morpholino)propanesulfonic acid (MOPS) running buffer. After electrophoresis, OBs were electroblotted onto a PVDF membrane in a Semi-Dry Transfer Cell (Bio-Rad). The resulting membranes were blocked overnight at 4°C in a solution containing 3% (w/v) BSA in TBS buffer, pH 7.4. Immunodetection of the OB-associated caleosin was carried out by incubation with a polyclonal Ab (diluted 1:500 in blocking solution) raised against the N-terminal (αN) sequence of Clo3 caleosin from *Arabidopsis thaliana *[22], for 12 h at 4°C. A goat anti-rabbit IgG conjugated with DyLight 549 (Agrisera AB, Sweden), diluted 1:2000 in TBST buffer, served as the secondary Ab. The signal was detected in a Pharos FX molecular imager (Bio-Rad), and images were recorded using the Quantity One v.4.6.2 software (Bio-Rad).

### Immunolocalization of caleosins in PEGylated OBs

Immunolocalization of caleosins was carried out on non-PEGylated and PEGylated OBs. For this purpose, OBs (40 μl per sample) were incubated in a 1.5 ml tube with either the FL or the αN anti-Clo3 Ab (diluted 1:30 in grinding medium), for 2 h at room temperature. Further, OBs were then incubated with an anti-rabbit IgG secondary Ab conjugated with DyLight 549 (Agrisera AB), diluted 1:200 in a 100 mM HEPES buffer (pH 7.5) containing 0.4 M sucrose, for 1 h at 37°C under gentle agitation. A few drops of an anti-fading solution of Citifluor (Sigma-Aldrich) were added and samples were observed in an Axioplan epifluorescence microscope (Carl Zeiss) under white and green light irradiation. Images were obtained in a ProGres C3 digital camera, using the ProGres CapturePro v2.6 software (Jenoptik AG).

## List of abbreviations

Ab: antibody; BSA: bovine serum albumin; DTT: dithiothreitol; EDTA: ethylenediaminetetraacetic acid; EGTA: ethylene-glycol-bis(β-aminoethylether)-*N, N, N', N'*-tetraacetic acid; ER: endoplasmic reticulum; HEPES: (4-(2-hydroxyethyl)-1-piperazineethanesulfonic acid); 13-HPOD: (9*Z*,11*E*,13*S*)-13-hydroperoxy octadeca-9,11-dienoic acid; OB: oil body; PBS: phosphate buffered saline; PCD: programmed cell death; PEG-MAL: polyethylene glycol-maleimide; PL: phospholipid; PMC: pollen mother cell; PVDF: polyvinylidene fluoride; PVPP: polyvinylpolypyrrolidone; TAG: triacylglycerol; TBS: Tris-buffered saline.

## Authors' contributions

AJC and MIRG conceived the study. AJC designed and supervised the experiments. KZ and AZ carried out the experiments. The four authors discussed the results and prepared the manuscript. All authors read and approved the final manuscript.
